# Optimization of odd chain fatty acid production by *Yarrowia lipolytica*

**DOI:** 10.1186/s13068-018-1154-4

**Published:** 2018-06-07

**Authors:** Young-Kyoung Park, Thierry Dulermo, Rodrigo Ledesma-Amaro, Jean-Marc Nicaud

**Affiliations:** 1 UMR1319, Team BIMLip: Biologie Intégrative du Métabolisme Lipidique, Institut Micalis, INRA-AgroParisTech, Université Paris-Saclay, Domaine de Vilvert, 78352 Jouy-en-Josas, France; 2 Lesaffre International, Marcq-en-Baroeul, France; 3 Department of Bioengineering, Imperial College London, London, UK

**Keywords:** *Yarrowia lipolytica*, Oleaginous yeast, Biolipid, Propionate, Odd chain fatty acids, Pentadecanoic acid, Heptadecanoic acid, Heptadecenoic acid, Metabolic engineering

## Abstract

**Background:**

Odd chain fatty acids (odd FAs) have a wide range of applications in therapeutic and nutritional industries, as well as in chemical industries including biofuel. *Yarrowia lipolytica* is an oleaginous yeast considered a preferred microorganism for the production of lipid-derived biofuels and chemicals. However, it naturally produces negligible amounts of odd chain fatty acids.

**Results:**

The possibility of producing odd FAs using *Y. lipolytica* was investigated. *Y. lipolytica* wild-type strain was shown able to grow on weak acids; acetate, lactate, and propionate. Maximal growth rate on propionate reached 0.24 ± 0.01 h^−1^ at 2 g/L, and growth inhibition occurred at concentration above 10 g/L. Wild-type strain accumulated lipids ranging from 7.39 to 8.14% (w/w DCW) depending on the carbon source composition, and odd FAs represented only 0.01–0.12 g/L. We here proved that the deletion of the *PHD1* gene improved odd FAs production, which reached a ratio of 46.82% to total lipids. When this modification was transferred to an obese strain, engineered for improving lipid accumulation, further increase odd FAs production reaching a total of 0.57 g/L was shown. Finally, a fed-batch co-feeding strategy was optimized for further increase odd FAs production, which generated 0.75 g/L, the best production described so far in *Y. lipolytica*.

**Conclusions:**

A *Y. lipolytica* strain able to accumulate high level of odd chain fatty acids, mainly heptadecenoic acid, has been successfully developed. In addition, a fed-batch co-feeding strategy was optimized to further improve lipid accumulation and odd chain fatty acid content. These lipids enriched in odd chain fatty acid can (1) improve the properties of the biodiesel generated from *Y. lipolytica* lipids and (2) be used as renewable source of odd chain fatty acid for industrial applications. This work paves the way for further improvements in odd chain fatty acids and fatty acid-derived compound production.

**Electronic supplementary material:**

The online version of this article (10.1186/s13068-018-1154-4) contains supplementary material, which is available to authorized users.

## Background

With the increasing environmental and energy concern, microbial oils (lipids and fatty acid-derived products) are regarded as promising alternatives to fossil fuels that can be used for the production of biofuels and oleo-chemicals. Microbial oils present multiple advantages over plant oils or animal fats, because they are not competitive with food, are less susceptible to seasonal availability, and they can be engineered to tune their composition and, therefore, properties [1]. For these reasons, many attempts have taken place to enhance lipid production from microorganisms with diverse metabolic engineering approaches. Despite the enhancement of microbial oils production, costs are not low enough to make the process economically feasible. One way to reduce production costs is to use low-cost carbon substrates [2]. Another is to produce value-added lipids or chemicals not readily obtainable via traditional petrochemical processes [3]. An example of this would be the production of odd chain fatty acids.

Microbial lipids contain mostly fatty acids from 12 to 22 carbon atoms, with the prevalence of the even-numbered of 16–20 [4]. The availability of fatty acids with odd carbon number is scarce, although they are more valuable for commercialization because of their various applications [3]. For example, *cis*-9-heptadecenoic acid is known to have an anti-inflammatory effect and actives on psoriasis, allergies, and autoimmune diseases [5]. Pentadecanoic acid and heptadecanoic acid can be used as biomarkers for dietary food intake assessment, coronary heart disease (CHD) risk, and type II diabetes mellitus risk [6–9]. The chemical properties and potential biological activities of odd chain fatty acids are now being more extensively studied [4], so novel nutritional and pharmaceutical application could be discovered. The biodiesel properties directly depend on the fatty acid composition of biodiesel fuel [10]. Importantly, and although most effect is caused by the saturated/unsaturated fatty acid ratio, odd chain fatty acids also have a positive impact in the quality of biofuels enhancing transesterification reactions or storage conditions. In addition to fuels, the odd chain fatty acids and derivatives are precursors for manufacturing agricultural chemicals like biocides, flavor and fragrance intermediates, hydraulic fluids, plasticizers, coatings, and industrial chemicals [11–14].

Despite the wide range of application, studies aimed to produce odd chain fatty acids in microorganisms are limited because most of microbial cells normally produce even chain fatty acids. It is reported that exogenous propionate (C3) can be used as a primer for synthesis of odd chain fatty acids: Propionate can be converted to propionyl-CoA by propionyl-CoA synthase, and propionyl-CoA is condensed with malonyl-CoA in the first step of odd chain fatty acid synthesis [15]. A metabolic engineering strategy with propionate supplementation achieved a production of 0.276 g/L odd chain free fatty acids in *E. coli* [16]. In addition, further engineering of *E. coli* showed an increased percentage of odd chain free fatty acids in total free fatty acids by 6.25-fold with propionate supplementation [17]. Odd chain fatty acids have also been produced, with propionate supplementation, in both oleaginous yeasts (*Candida* sp., *Rhodotorula glutinis*, *Trichosporon cutaneum*, *Y. lipolytica*, *Cryptococcus curvatus*) and non-oleaginous yeast (*Kluyveromyces polysporus*, *Saccharomyces cerevisiae*, *Torulaspora delbrueckii*) [18, 19]. In the best performing yeast, *Y. lipolytica*, odd chain fatty acid did not exceed about 30% of total lipid with a maximum total lipid production of 0.31 g/L, and the highest lipid content of 8.9% g/g of cell dry weight (CDW) [18]. The studies on odd chain fatty acids production in yeast have been done, so far, by optimizing fermentation conditions [19] or evaluating capacity of producing lipids among several strains [18]. More research on propionate utilization and metabolic engineering approaches for enhancing odd chain fatty acids production are, therefore, needed.

Propionic acid is an abundant volatile fatty acid (VFA) which can be obtained from agro-industrial lignocellulosic wastes, sludge and several biodegradable organic wastes [20]. Recently, VFAs is gaining interests as a substrate for lipid production by oleaginous microorganisms since it can be produced from wastes with low-costs [18, 21, 22]. Additionally, VFAs including propionate could lead to higher theoretical conversion efficiencies to lipids compared to other sugar-based carbon sources such as glucose and glycerol due to their shorter metabolic pathways [19, 23]. Studies on tolerance and utilization of propionic acid by oleaginous yeast for lipid production are still limited in the literature, it is sure that further research are necessary to use VFAs, either propionate alone or mixture of VFAs, as more feasible carbon sources.

*Yarrowia lipolytica* is a widely recognized oleaginous yeast known for its superior characteristics in the production of lipids and fatty acid-derived compounds, as well as other biotechnological products such as organic acids, nutraceuticals, emulsifiers, and surfactants [2, 24]. In addition, *Y. lipolytica* can grow in a broad range of substrates and it has been recently engineered for expanding the substrates range of this yeast including renewable biomass. Several strategies by overexpressing genes or deleting competitive pathway have also been used for improving even lipid accumulation in *Y. lipolytica* [25–27].

In this work, we investigated the ability of *Y. lipolytica* to produce odd chain fatty acids from propionate either as sole carbon and energy source or in combination with glucose. To increase the propionyl-CoA pool for the synthesis of odd chain fatty acids, we disrupted *PHD1* encoding 2-methylcitrate dehydratase in the methyl citrate cycle. We also engineered the strain to accumulate more fatty acid by enhancing the synthesis capacity and blocking the degradation of lipids. Additionally, a fed-batch co-feeding strategy with glucose and propionate further increased total odd chain fatty acids. This work paves the way to use *Y. lipolytica* as a platform microorganism for producing valuable biochemicals with odd-numbered carbon chain.

## Results and discussion

### *Yarrowia lipolytica* can grow on propionate as sole carbon source

**Fig. 1:**
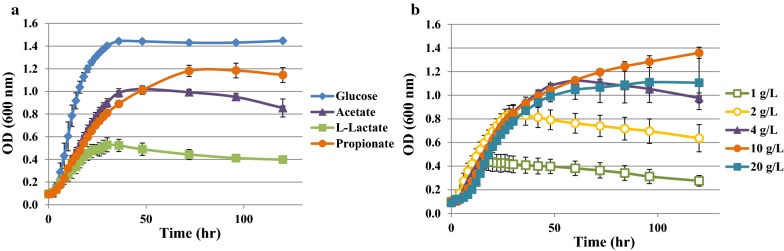
Growth of wild-type W29 derivative strain JMY2900 on weak acids and on different propionate concentration. **a** Cell growth in minimal media containing 5 g/L of glucose, acetate, l-lactate and propionate as a sole carbon source. **b** Cell growth in propionate medium from 1 to 20 g/L. Growth curves are representative of three independent tests

It is known that *Y. lipolytica* can be grown using diverse carbon sources from hydrophobic substrates such as *n*-alkanes, fatty acids, and oils to hydrophilic ones such as sugars or organic acids [21]. It was also reported that VFAs could be used as substrates for lipid production [18, 21, 28]. However, high concentration of VFA and weak acids inhibit cell growth which differs depending on strains [18, 21, 28, 29]. The *Y. lipolytica* strains used in this study were derived from the wild-type *Y. lipolytica* W29 strain (ATCC20460). The auxotrophic derivative Po1d (Leu− Ura−, Table 1, Additional file 1: Figure S1) was previously described by Barth and Gaillardin [30]. The Po1d prototroph derivative JMY2900 (Table 1) was used as wild-type reference strain for the comparison with engineered strains derived from Po1d [31]. Growth performance of our reference strain on weak acids and inhibitory effect of propionate were analyzed in microplate (Fig. 1). *Y. lipolytica* was able to grow on weak acids at similar growth rate, about 0.16 h^−1^, lower than in glucose (0.25 h^−1^) (Fig. 1a, Additional file 1: Table S1). As previously shown, *Y. lipolytica* can utilize propionate as a sole carbon source—a substrate that promotes odd chain fatty acid production. Our reference *Y. lipolytica* strain JMY2900 was able to grow on propionate although the growth rate and the final OD were lower than in glucose (Fig. 1a). This growth inhibition was also shown in other organic acids in the following order: l-lactate > propionate > acetate. In comparison to acetate, growth on propionate showed lower growth rate (0.16 h^−1^) but higher final OD at same concentration (5 g/L). Although acetate has been regarded as preferable carbon source among VFAs because of their relatively lower growth inhibitory effect in previous studies [21, 28], our results showed that propionate can be also a potential carbon source for biomass and lipid production in our *Y. lipolytica* strain.

**Table 1:** *E. coli* and *Y. lipolytica* strains used in this study

Name	Relevant genotype/plasmid description	Source of references
*E. coli* strains		
DH5α	$\varPhi$80*lacZ*∆m15 ∆(*lac*ZYA-*arg*F) U169 *rec*A1 *end*A1 *hsd*R17 (r_k_^−^, m_k_^+^) *pho*A *sup*E44 *thi*-1 *gyr*A96 *rel*A1 λ^−^	Promega
JME740	DH5α pKS-PUT *phd1*	[32]
JME547	DH5α p*UB4*-*CRE1*	[53]
JME1000	DH5α pKS-PLT *tgl4*	[37]
JME1077	DH5α pGEMT easy-PUT *mfe1*	[36]
JME1128	DH5α JMP62-pTEF-*GPD1*-*URA3*ex	[36]
JME1822	DH5α JMP62-pTEF-*DGA2*-*LEU2*ex	[38]
*Y. lipolytica* strains		
Po1d (JMY195)	*MAT*a *ura3*-*302 leu2*-*270 xpr2*-*322*	[30]
JMY2900	Po1d Ura+ Leu+	[31]
JMY1203	Po1d *phd1::URA3*ex	[32]
JMY3279	Po1d *∆phd1*	This study
JMY3348	Po1d *∆phd1 mfe1::URA3ex*	This study
JMY3350	Po1d *phd1::URA3ex *+* LEU2*	This study
JMY3396	Po1d *∆phd1 mfe1::URA3ex tgl4::LEU2ex*	This study
JMY3433	Po1d *∆phd1 ∆mfe1 ∆tgl4*	This study
JMY3576	Po1d *∆phd1 ∆mfe1 ∆tgl4* +*pTEF*-*DGA2*-*LEU2ex*	This study
JMY3776	Po1d *∆phd1 ∆mfe1 ∆tgl4* +*pTEF*-*DGA2*-*LEU2ex *+ *pTEF*-*GPD1*-*URA3ex*	This study

In a previous study, it is reported that propionate has an inhibitory effect on the cell growth at concentrations above 5 g/L [21]. To explore if our strain could grow on higher concentration than 5 g/L, JMY2900 was cultivated with different concentrations of propionate (Fig. 1b, Additional file 1: Figure S2). Our strain was able to grow up to 100 g/L of propionate as a sole carbon source, the highest growth rate was observed at 2 g/L of propionate (Additional file 1: Table S2). There was no big difference in initial OD trends between 4 and 10 g/L of propionate, but JMY2900 on 10 g/L of propionate was able to grow at higher cell density. The inhibitory effect of propionate to the cell growth was observed on concentration higher than 10 g/L (Additional file 1: Table S2). The growth test at higher concentration of propionate (100 g/L) showed a long lag phase of more than 48 h (Additional file 1: Figure S2), this shows propionate can be used as a carbon source in our strain. However, several *Y. lipolytica* strains behave very differently. For example, our strain appeared to be less sensitive to propionate than *Y. lipolytica* strain CICC31596 which shows an inhibitory effect of propionate on growth rate and lag phase already at 5 g/L [28], while *Y. lipolytica* strain ISA 1834 showed higher growth rate, 0.29 h^−1^, on propionate [29]. This demonstrates important differences in propionate sensitivity depending on either strains, culture conditions or media composition.

### Accumulation of odd chain fatty acids in propionate medium

**Fig. 2:**
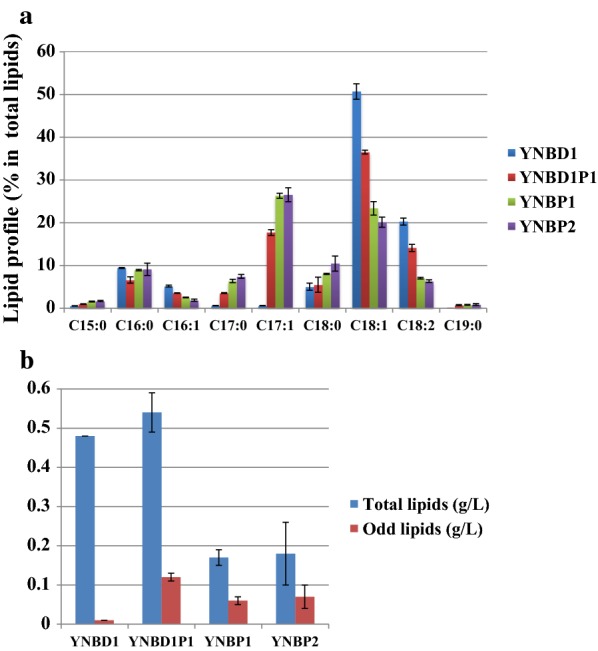
Lipid profiles and lipid production in WT strain on different media. JMY2900 was grown in glucose (YNBD1), glucose and propionate (YNBD1P1), and in propionate media (YNBP1 and YNBP2) for 72 h at 28 °C, 180 rpm. The results presented are the mean values ± SD for three independent biological replicates. **a** Lipid profiles depending on carbon sources. **b** Total lipid (blue) and total odd lipid (red) production

To see whether propionate is a suitable carbon source for odd lipid production, flask cultures of JMY2900 with several compositions of carbon sources (YNBD1 glucose 1%, YNBD1P1 glucose 1% and propionate 1%, YNBP1 propionate 1%, YNBP2 propionate 2%) were carried out. It was revealed that lipid content obtained on YNBP1 (7.48%) was comparable to that on YNBD1 (7.86%) with significant difference of lipids composition (Table 2, Fig. 2). In YNBD1 media, oleic acid (C18:1) and linoleic acid (C18:2) were the major products with a percentage of 50.72 and 20.26%, respectively. Only 1.75% of odd chain fatty acids in total fatty acids were produced in this condition. However, in case of medium without glucose like YNBP1 and YNBP2, the ratio of odd chain fatty acid to total lipids increased to around 35%. In these conditions, the portion of oleic acid and linoleic acid in total lipids decreased in contrast to the increase of heptadecenoic acid (C17:1). In addition to heptadecenoic acid (C17:1), other odd chain fatty acids such as pentadecanoic acid (C15:0), heptadecanoic acid (C17:0), and nonadecanoic acid (C19:0) were also produced from all propionate-containing medium (Table 2, Fig. 2a). These results indicate that propionate can be used as a primer for the synthesis of odd chain fatty acids in *Y. lipolytica* as reported in other studies [18, 19]. Although the total lipid contents from YNBD1 and YNBP1 are similar, the biomass produced was significantly different (5.37 and 2.60 g/L, respectively). The difference in biomass production in YNBP1 and YNBP2 was already shown in Fig. 1b, and it might be due to a higher the inhibitory effect of higher concentration of propionate. In spite of lower ratio of odd chain fatty acids to total fatty acids in YNBD1P1 than that of YNBP1, 0.12 g/L of odd chain fatty acid was produced which showed the highest amount in this culture (Table 2). JMY2900 accumulated slightly higher odd and total lipids in YNBP2 than YNBP1, but it did not show significantly better performance for odd chain fatty acids production. In addition, higher concentration of propionate showed inhibitory effect from the beginning of culture (data not shown). From these results, YNBD1P1 is the best condition for the odd and total lipids production, and YNBP1 is also a suitable condition for high ratio of odd chain fatty acids to total fatty acids (Fig. 2b).

**Table 2:** Biomass and lipid production by wild-type strain JMY2900 in minimal medium

	Biomass (g/L)	Lipid content % (g/g dry cell)		Odd lipids/total lipids (%)	Lipids (g/L)	
		Total	Odd		Total	Odd
YNBD1	5.37 ± 0.13	7.86 ± 0.13	0.14 ± 0.00	1.75 ± 0.02	0.48 ± 0.00	0.01 ± 0.00
YNBD1P1	6.80 ± 0.08	7.39 ± 0.15	1.70 ± 0.06	22.93 ± 0.68	0.54 ± 0.05	0.12 ± 0.01
YNBP1	2.60 ± 0.37	7.48 ± 0.69	2.61 ± 0.21	34.96 ± 0.45	0.17 ± 0.02	0.06 ± 0.01
YNBP2	2.71 ± 0.58	8.14 ± 1.51	3.01 ± 0.72	36.52 ± 2.18	0.18 ± 0.08	0.07 ± 0.03

Strain was grown for 72 h at 28 °C and 180 rpm

The mean value of three independent experiments is shown and the standard deviation is indicated

### Inactivation of the propionate catabolic pathway improved odd chain fatty acid content and production

**Fig. 3:**
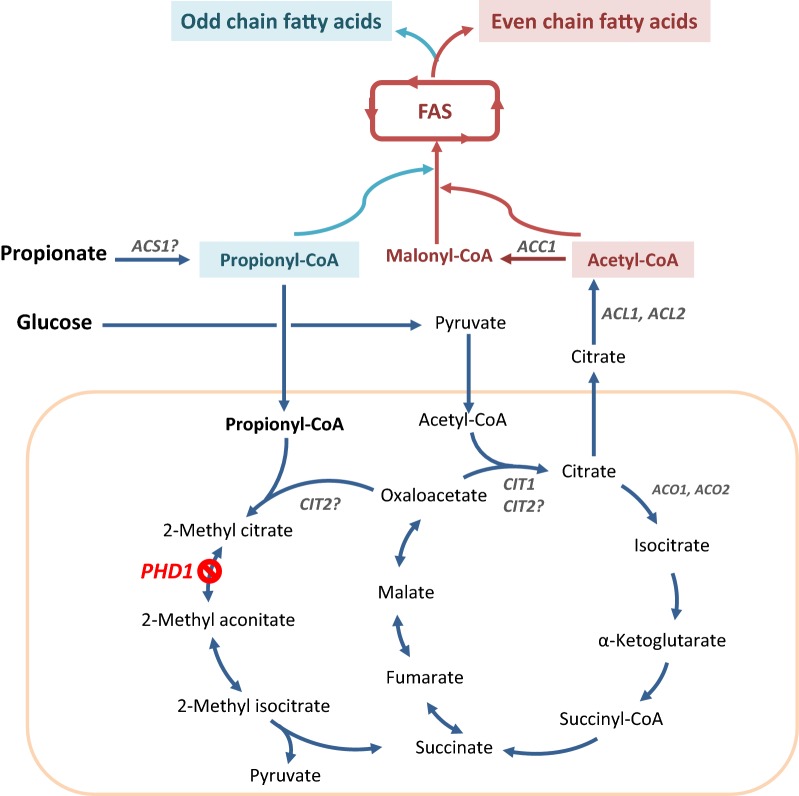
Overview of the pathways involved in odd and even fatty acid production including the link with the TCA and methyl citrate cycles in *Yarrowia lipolytica*. Propionate is activated by the cytosolic acyl-CoA synthase (*ACS1*) to form the propionyl-CoA which is transported into mitochondria to enter the methyl citrate pathway. Propionate is condensed with oxaloacetate to form 2-methyl citrate by 2-methylcitrate synthase probably encoded by *CIT2* (*CIT2*, YALI0E02684g). 2-Methyl citrate dehydratase removes an H_2_O to form 2-methyl aconitate by 2-methyl citrate dehydratase (*PHD1*, YALI0F02497), then is hydrated to form 2-methyl isocitrate probably by the aconitase (*ACO1*, YALI0D09361g; *ACO2*, YALI0E14949g), which is cleaved by 2-methyl isocitrate lyase (YALI0F31999g) to give succinate and pyruvate. Glucose undergoes glycolysis and enters the mitochondria as a form of pyruvate to be used in the TCA cycle. Mitochondrial pyruvate is condensed with oxaloacetate by citrate synthase (*CIT1*, YALI0E00638g) to form citrate which can be exported to cytosol. The cytosolic citrate is transformed by the ATP-citrate lyase (subunit a, *ACL1*, YALI0E34793g and subunit b, *ACL2*, YALI0D24431g) into acetyl-CoA. Acetyl-CoA is then converted into malonyl-CoA by the acetyl-CoA carboxylase (*ACC1*, YALI0C11407g) as the first step of fatty acid synthesis. Acetyl-CoA and malonyl-CoA are condensed by the fatty-acid synthase complex (FAS; subunit beta, *FAS1*, YALI0B15059g and subunit alpha, *FAS2*, YALI0B19382g) for the production of even fatty acids, while acetyl-CoA and propionyl-CoA are condensed for the production of odd fatty acids

**Fig. 4:**
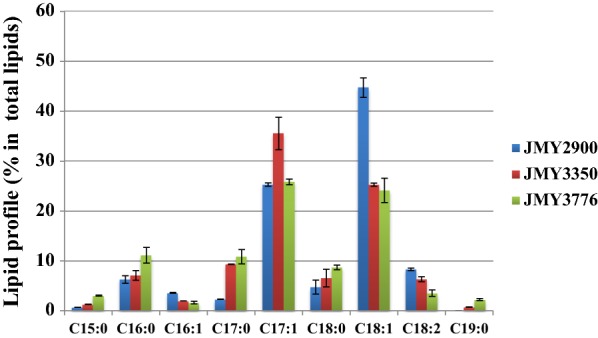
Comparison of lipid profiles of JMY2900 (WT), JMY3350 (WT ∆*phd1*), and JMY3776 (obese ∆*phd1)* in glucose and propionate media. Cells were grown for 72 h at 28 °C, 180 rpm. The results presented are the mean values for three independent biological replicates

We previously reported the importance of the methylcitrate cycle on glycerol metabolism in *Y. lipolytica* [32]. *PHD1*, involved in the synthesis of 2-methylcitrate dehydratase is a mitochondrial protein, which catalyzes the conversion of 2-methyl citrate to 2-methyl-*cis*-aconitate in the methyl citrate cycle. It has been shown that, in *Y. lipolytica*, the deletion of *PHD1* results in the accumulation of 2-methyl citrate, which could potentially halt the TCA cycle and inhibit the entry of citrate into mitochondria [32]. Additionally, deletion of the *PHD1* gene coding for the 2-methyl-citrate dehydratase was shown to improved lipid accumulation. As described above, propionate can be converted to propionyl-CoA, which promotes the production of odd chain lipids. In *Y. lipolytica*, propionyl-CoA can be catabolized to form pyruvate and succinate through the methyl citrate cycle (Fig. 3). So the methyl citrate cycle can be regarded as competitive pathway for the synthesis of odd chain fatty acids. We hypothesize here that inhibition of 2-methyl citrate pathway by deleting *PHD1* increases the propionyl-CoA pool that could be used for further synthesis of odd chain fatty acids. To prove this, culture of JMY3350 (WT ∆*phd1*) was performed in the same condition as above. As expected, JMY3350 was not able to grown in YNBP1 since that propionate cannot be used as a sole carbon source. This confirmed that propionate cannot be metabolized through methyl citrate cycle to form pyruvate in JMY3350. In glucose media (YNBD1), we observed an increased of the ratio of odd chain fatty acid to total fatty acid even without propionate by 1.35 times (Additional file 1: Table S3). Inactivation of *PHD1* blocks the TCA cycle [32], which might cause growth defects and increase sensitivity of propionate to the cell. Therefore, we added lower amount of propionate (4 g/L) with glucose (10 g/L) after 16 h of the start of the culture with glucose (YNBD1). To compare the capability of the two strains, JMY2900 (WT) and JMY3350 (WT ∆*phd1*), for odd chain fatty acid production, we used an equivalent amount of metabolizable carbon with a C/N ratio 30, which is often found as the optimum condition for lipid production in *Y. lipolytica* [33–35]. Therefore, the glucose amount was adjusted in JMY3350 strain to compensate the lack of use of propionate as carbon source for biomass formation. The lipid content of JMY3350 increased by 17% comparing with the wild-type (8.01 and 6.85%). The ratio of odd chain fatty acids to total lipids was also higher (46.82%) than the wild-type (28.32%) (Table 3). JMY3350 produced 0.17 g/L of odd chain fatty acids 21.4% higher than control strain despite of its lower biomass. The lower biomass formation has been previously reported for this mutant [32]. In addition, the inactivation of *PHD1* modified the composition of lipids (Fig. 4). The percentage of heptadecanoic acid in total lipids increased 4 times. Heptadecenoic acid (C17:1) showed the highest portion (35.56%) of total lipids in WT ∆*phd1* (JMY3350), while JMY2900 produced mostly oleic acid (44.75%), likewise most of other *Y. lipolytica* strains. Stability of modified strains is a key parameter in a bioprocess. In this regard, ∆*phd1* strains is expected to be stable on time, since the gene was completely removed from genome and it is very unlikely that other enzymes evolve to consume propionate in a fermentation condition when glucose is the fed for growing.

**Table 3:** Biomass and lipid production by WT, WT ∆*phd1* and obese ∆*phd1* strains in minimal glucose and propionate media

	Biomass (g/L)	Lipid content % (g/g dry cell)		Odd/total lipids (%)	Lipids (g/L)	
		Total	Odd		Total	Odd
JMY2900 (WT)	7.18 ± 0.25	6.85 ± 0.21	1.94 ± 0.03	28.32 ± 0.01	0.49 ± 0.01	0.14 ± 0.01
JMY3350 (WT ∆*phd1*)	4.50 ± 0.50	8.01 ± 0.64	3.75 ± 0.04	46.82 ± 0.03	0.36 ± 0.01	0.17 ± 0.02
JMY3776 (obese ∆*phd1*)	5.53 ± 0.32	24.76 ± 2.51	10.37 ± 0.49	41.9 ± 0.02	1.36 ± 0.06	0.57 ± 0.01

Strain JMY2900 (WT), JMY3350 (WT ∆*phd1*) and JMY3776 (obese ∆*phd1)* were grown for 72 h at 28 °C and 180 rpm

The mean value of three independent experiments is shown and the standard deviation is indicated

### Engineering higher accumulation of odd chain FA

Once we verified that strain carrying deletion of *PHD1* was able to produce more odd chain fatty acids, we wanted to engineer the strain to make it able to accumulate higher amount of total odd chain lipids. Therefore, we generated the strain named obese ∆*phd1* (JMY3776) by multiple modifications (Additional file 1: Figure S1). First, to block β-oxidation, *MFE1* encoding the multifunctional enzyme, involved in the second step of β-oxidation, was deleted [36]. To inhibit triacylglycerols (TAG) remobilization, *TGL4* encoding a triglyceride lipase, was deleted [37]. In addition, to push and pull TAG biosynthesis, *DGA2* encoding the major acyl-CoA: diacylglycerol acyltransferase [25, 38], and *GPD1* encoding glycerol-3-phosphate dehydrogenase were overexpressed [36]. We then studied lipid production of engineered strain JMY3776 in the same conditions as before. As expected, odd chain lipid accumulation increased as well as total lipid accumulation, by 3.35 and 3.78 times, respectively (Table 3). The ratio of odd chain lipids to total lipids was slightly decreased in JMY3776, but still remained above 40%. The amount of odd chain fatty acids was 0.57 g/L, the highest amount produced in *Y. lipolytica*, so far. The amount of all saturated fatty acids from C15:0 to C19:0 increased all together contrary to the unsaturated fatty acids moiety, which decreased (Fig. 4). This phenomenon was also shown in our previous study [39], the strains optimized for lipid accumulation (called obese strain) produced more C16:0 than wild-type and less unsaturated C16 and C18 commonly in different carbon sources (glucose, fructose, and sucrose).

### Increase of accumulation of odd FA by fed-batch co-feeding carbon sources

As described above, propionate is a key carbon source for production of odd lipids meanwhile shows growth inhibitory effect in *Y. lipolytica*. Besides, the engineered strain is more sensitive to propionate allowing only small amount of propionate being used for odd chain lipid synthesis. Several fed-batch fermentation strategies have been used to improve yield and productivity by avoiding high level of inhibitory compounds in culture medium [40, 41]. To see whether fed-batch strategy could boost production of odd chain lipids while minimizing the inhibitory effect of propionate, we investigated fed-batch co-feeding of carbon sources during cultivation.

**Fig. 5:**
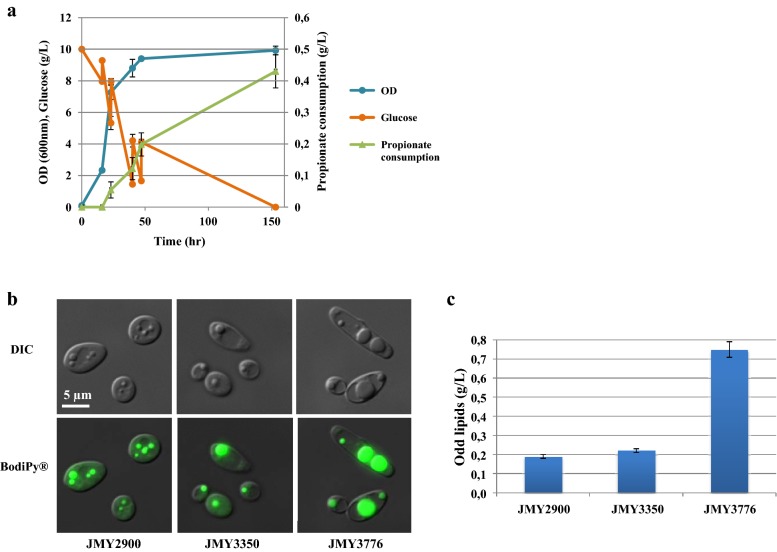
Improvement of odd chain fatty acid production of JMY3776 by fed-batch co-feeding of propionate and glucose. **a** Increase of the accumulation of odd lipids by co-feeding of propionate and glucose. Cells were grown for 16 h in glucose media (YNBD1), then pulses of glucose + propionate (4 and 0.5 g/L, respectively) were added at *t* = 16, 23, 40, 47 h. The results presented are the mean values for three independent biological replicates. **b** Microscope image of cells (DIC) and lipid body visualization with Bodipy of JMY2900 (WT), JMY3350 (WT ∆*phd1*) and JMY3776 (obese ∆*phd1*). **c** Increase of odd lipids accumulation by pathway engineering in fed-batch co-feeding cultures

The obese ∆*phd1* strain was cultured in YNBD1 with addition of carbon sources (glucose 4 g/L and propionate 0.5 g/L) at four times points (Fig. 5a). As a result, the production of total lipid and odd chain lipid content, compare to batch culture, were increased by 50.35 and 12.64%, respectively (Table 4). However, the percentage of odd chain lipids in total lipids is diminished by 60% as compared to batch condition likely due to the co-feeding with glucose. Nevertheless, the amount of total odd chain fatty acids from fed-batch co-feeding reached 0.75 g/L, 31% higher than in batch, which represents the highest titer produced in *Y. lipolytica* so far (Additional file 1: Table S4). This represents a 395% increase of odd chain fatty acid production between wild-type JMY2900 and the obese ∆*phd1* deleted strain in the fed-batch condition (Fig. 5b, c). Fed-batch fermentation has been beneficial for the production of other compounds by *Y. lipolytica*, such as the production of lipids described for the obese strain JMY3501 on synthetic media [42] or the obese strain producing carotenoides with the concomitant production of 42.6 g/L of lipids on rich media [43]. The fermentation conditions can be further optimized by testing various feeding rate of glucose and propionate, which will allow to improve final biomass, higher lipid content and odd chain fatty acid content. Also, in future experiments it would be interesting to test other C/N ratios, such as C/N = 60 or C/N = 100 which are found better for certain strains and conditions [25, 39, 44, 45].

**Table 4:** Biomass and lipid production by WT, WT ∆*phd1* and obese ∆*phd1* strains by fed-batch co-feeding of glucose and propionate

	Biomass (g/L)	Lipid content % (g/g dry cell)		Odd/total lipids (%)	Lipids (g/L)	
		Total	Odd		Total	Odd
JMY2900 (WT)	8.20 ± 0.05	17.23 ± 0.31	2.30 ± 0.02	13.36 ± 0.01	1.41 ± 0.02	0.19 ± 0.01
JMY3350 (WT ∆*phd1*)	4.83 ± 0.13	17.71 ± 0.70	4.58 ± 0.05	25.90 ± 0.01	0.85 ± 0.01	0.22 ± 0.01
JMY3776 (obese ∆*phd1*)	5.93 ± 0.13	50.35 ± 1.99	12.64 ± 0.45	25.11 ± 0.17	2.99 ± 0.18	0.75 ± 0.04

Additionally, one could envisage the production of different types of odd chain fatty acids and their derivatives such as shorter odd chain fatty acid by engineering the fatty acid synthase (FAS) as recently demonstrated in *Yarrowia lipolytica* [46], hydroxylated odd chain fatty acid by introducing ∆12-hydroxylase (FAH12) from *Claviceps purpurea* [47] and odd chain dicarboxylic acid by overexpression of the omega oxidation pathway [48]. However, further basic knowledge is required to fully understand propionic acid catabolism, its transport and toxicity to the cells.

## Conclusion

In this study, it is shown that *Y. lipolytica* produce odd chain fatty acids (mainly heptadecenoic acid, heptadecanoic acid, and pentadecanoic acid) from propionate as a carbon source. By inactivating 2-methyl-citrate dehydratase in competing pathway utilizing propionyl-CoA, the amount of odd chain fatty acids is increased, the ratio of odd lipid to total lipids reached to 46.82%. Obese ∆*phd1* strain engineered to accumulate higher amount of lipid produced 3.35 times higher odd chain lipids together with increased total lipid accumulation. In addition, a fed-batch co-feeding strategy further improved production of odd chain fatty acids with amount of 0.75 g/L represents the highest titer produced in *Y. lipolytica* so far. Odd chain fatty acids are very important and versatile chemicals in both pharmaceutical and industrial fields. This work paves the way for further improvements in odd chain fatty acids and fatty acid-derived compound production.

## Methods

### Strains and media

Media and growth conditions for *E. coli* were as described by Sambrook et al. [49], and those for *Y. lipolytica* have been described by Barth and Gaillardin [30]. Rich medium (YPD) and minimal glucose medium (YNB) were prepared as described previously [50]. Minimal medium (YNB) contained 0.17% (w/v) yeast nitrogen base (without amino acids and ammonium sulfate, YNBww, Difco), 0.5% (w/v) NH_4_Cl, 50 mM phosphate buffer (pH 6.8). The following carbon sources were added: YNBD1 (1% (w/v) glucose, YNBD1P1 (1% (w/v) glucose, 1% (w/v) propionate), YNBP1 (1% (w/v) propionate), YNBP2 (2% (w/v) propionate). To complement auxotrophic processes, 0.1 g/L of uracil or leucine (Difco) was added as necessary.

### Construction of strains

The overexpression and disruption cassettes were prepared as described previously [36–38], and were used for transformation by the lithium acetate method [51]. Transformants were selected on YNBUra, YNBLeu, YNBHyg, YNB media depending on their genotype. Then genomic DNA from yeast transformants was prepared as described in Querol et al. [52]. Positive transformants were checked by PCR. The removal of the selection marker was carried out via the LoxP-Cre system as previously used in *Y. lipolytica* [53].

Restriction enzymes were obtained from New England Biolabs (Ipswich, MA, USA). PCR amplifications were performed in an Eppendorf 2720 thermal cycler with GoTaq DNA polymerases (Promega) and Q5 High-Fidelity DNA Polymerase (New England Biolabs). PCR fragments were purified with a QIAgen Purification Kit (Qiagen, Hilden, Germany).

### Growth test

Pre-cultures were inoculated into tubes containing 5 mL YPD medium, and cultured overnight (28 °C, 180 rpm). Pre-cultures were then centrifuged and washed with sterile distilled water, cell suspensions were standardized to an OD_600_ of 0.1. Stains were grown in 200 μL of minimal YNB medium (see above) in the presence of carbon sources (0.5% glucose, propionate, l-lactate, acetate as a carbon source) in 96-well plates, with constant shaking, at 28 °C. Growth was monitored by measuring the optical density (OD_600_) every 30 min for 120 h with a microtiter plate reader (Biotek Synergy MX, Biotek Instruments, Colmar, France). For each strain and set of conditions, we used three biological replicates. The growth rate was calculated in the exponential phase for each strain and condition.

### Culture conditions for lipid biosynthesis experiments

For lipid biosynthesis in minimal media, cultures were prepared as follows: an initial pre-culture was established by inoculating 10 mL of YPD medium in 50 mL Erlenmeyer flasks. This was followed by an overnight shaking step at 28 °C and 180 rpm. The resulting cell suspension was washed with sterile distilled water and used to inoculate 50 mL of YNB medium containing 0.15% (w/v) NH_4_Cl and 50 mM phosphate buffer (pH 6.8) with various concentrations of carbon source in 250 mL Erlenmeyer flasks, at 28 °C and 180 rpm. For fed-batch co-feeding test, the strains were cultured in 20 mL of YNBD1 with addition 2 mL of mixture of carbon sources to a final concentration of glucose 4 g/L and propionate 0.5 g/L at four times points. The addition were performed at *T* = 16, 23, 40, 48 h after the inoculation.

### Determination of glucose and propionate

Glucose and propionate were identified and quantified by HPLC. Filtered aliquots of the culture medium were analyzed by UltiMate 3000 system (Thermo Fisher Scientific, UK) using an Aminex HPX-87H column (300 mm × 7.8 mm, Bio-RAD, USA) coupled to UV (210 nm) and RI detectors. The mobile phase used was 0.01 N H_2_SO_4_ with a flow rate of 0.6 mL/min and the column temperature was *T* = 35 °C. Identification and quantification were achieved via comparisons to standards. For each data point, we used at least two biological replicates and calculated average and standard deviation values.

### Lipid determination

Lipids were extracted from 10 to 20 mg of freeze-dried cells, and converted into their fatty acid methyl esters (FAMEs) according to Browse et al. [54], and FAMEs were analyzed by gas chromatography (GC) analysis. GC analysis of FAMEs was carried out on a Varian 3900 instrument equipped with a flame ionization detector and a Varian FactorFour vf-23 ms column, where the bleed specification at 260 °C is 3 pA (30 m, 0.25 mm, 0.25 μm). Fatty acids were identified by comparison to commercial FAME standards (FAME32, Supelco) and quantified by the internal standard method, involving the addition of 100 μg of commercial dodecanoic acid (Sigma-Aldrich). Commercial odd chain fatty acids (9 Odd carbon fatty acids, OC9, Supelco) were converted to their FAMEs with a same method for yeast samples, and analyzed by GC to identify and compare odd chain fatty acids from yeast samples.

To determine DCW in flask experiments, 2 mL of the culture were washed and lysophilized in a pre-weighed tube. The differences in weight corresponded to the mg of cells found in 2 mL of culture. For each data point, we used at least two biological replicates and calculated average and standard deviation values.

### Microscopic analysis

Images were obtained using a Zeiss Axio Imager M2 microscope (Zeiss, Le Pecq, France) with a 100× objective lens and Zeiss filter sets 45 and 46 for fluorescence microscopy. Axiovision 4.8 software (Zeiss, Le Pecq, France) was used for image acquisition. To make the lipid bodies (LBs) visible, BodiPy^®^ Lipid Probe (2.5 mg/mL in ethanol, Invitrogen) was added to the cell suspension (OD_600_ = 5) and the samples were incubated for 10 min at room temperature.

## Additional file

**Additional file 1: Figure S1.** Schematic representation of strain construction. The auxotrophic strain Po1d (Leu − Ura −) was derived from the French wild-type strain W29. First, *PHD1* was disrupted with the *phd1*::*URA3*ex disruption cassette (JME740) yielding JMY1203 and JMY3350 after *LEU2* complementation. Uracil auxotrophe was restored by marker rescue yielding JMY3279. Second, *MFE1* and TGL4 were disrupted with the *mfe1*::*URA3*ex disruption cassette (JME1077) yielding JMY3348 and the *tgl4*::*LEU2*ex disruption cassette (JME1000) yielding JMY3396. Uracil and leucine auxotrophies were restored by marker rescue yielding JMY3433. Finally, p*TEF*-*DGA2*-*LEU2ex* (JME 1822) and p*TEF*-*GPD1*-*URA3ex* (JME1128) overexpression cassettes were introduced yielding JMY3776 (obese ∆*phd1*). For more information about construction, see “Methods” section and Table 1. **Figure S2.** Cell growth in different concentration of propionate. **Figure S3.** GC chromatogram of lipid profiles of JMY2900 (WT) and JMY3776 (obese ∆*phd1*). **Table S1.** Growth rate of JMY2900 on glucose and weak acids. **Table S2.** Growth rate of JMY2900 on propionate depending on concentration. **Table S3.** Lipid production of JMY2900 and JMY3350 in YNBD1. **Table S4.** Odd chain Fatty acid production in *Y. lipolytica.*

## Abbreviations

DCW: dry cell weight
DIC: differential interference contrast
Odd FA: odd chain fatty acid
Even FA: even chain fatty acid
PCR: polymerase chain reaction
