# Supplementary material for: Optimization of odd chain fatty acid production by Yarrowia lipolytica
Source: Biotechnol Biofuels. 2018 Jun 7;11:158. doi: 10.1186/s13068-018-1154-4 (PMC5991449; doi:10.1186/s13068-018-1154-4)
Supplement: Supplementary file 1 — Additional file 1: Figure S1. Schematic representation of strain construction. The auxotrophic strain Po1d (Leu − Ura −) was derived from the French wild-type strain W29. First, PHD1 was disrupted with the phd1::URA3ex disruption cassette (JME740) yielding JMY1203 and JMY3350 after LEU2 complementation. Uracil auxotrophe was restored by marker rescue yielding JMY3279. Second, MFE1 and TGL4 were disrupted with the mfe1::URA3ex disruption cassette (JME1077) yielding JMY3348 and the tgl4::LEU2ex disruption cassette (JME1000) yielding JMY3396. Uracil and leucine auxotrophies were restored by marker rescue yielding JMY3433. Finally, pTEF-DGA2-LEU2ex (JME 1822) and pTEF-GPD1-URA3ex (JME1128) overexpression cassettes were introduced yielding JMY3776 (obese ∆phd1). For more information about construction, see “Methods” section and Table 1. Figure S2. Cell growth in different concentration of propionate. Figure S3. GC chromatogram of lipid profiles of JMY2900 (WT) and JMY3776 (obese ∆phd1). Table S1. Growth rate of JMY2900 on glucose and weak acids. Table S2. Growth rate of JMY2900 on propionate depending on concentration. Table S3. Lipid production of JMY2900 and JMY3350 in YNBD1. Table S4. Odd chain Fatty acid production in Y. lipolytica. [file 13068_2018_1154_MOESM1_ESM.docx]

**Additional materials**

*Biotechnology for Biofuels*Section: *Research*

***Optimization of odd chain fatty acid production by Yarrowia lipolytica***

**Young-Kyoung Park ^a^, Thierry Dulermo ^a,b^, Rodrigo Ledesma-Amaro ^a,c*^ and Jean-Marc Nicaud ^a*^**

^a^Micalis Institute, INRA, AgroParisTech, Université Paris-Saclay, Jouy-en-Josas, France

^b^Lesaffre International, Marcq-en-Baroeul, France

^c^Department of Bioengineering, Imperial College London, London, United Kingdom

E-mail address:

Young-Kyoung Park: [YoungKyoung.Park@inra.fr](mailto:YoungKyoung.Park@inra.fr)

Thierry Dulermo : [TYD@lesaffre.fr](mailto:TYD@lesaffre.fr)

Jean-Marc Nicaud: [jean-marc.nicaud@inra.fr](mailto:jean-marc.nicaud@inra.fr)

Rodrigo Ledesma-Amaro: [r.ledesma-amaro@imperial.ac.uk](mailto:r.ledesma-amaro@imperial.ac.uk)

***Corresponding authors**: Jean-Marc Nicaud and Rodrigo Ledesma-Amaro, Institut Micalis, INRA-AgroParisTech, UMR1319, Team BIMLip: Biologie Intégrative du Métabolisme Lipidique, Domaine de Vilvert, 78352 Jouy-en-Josas France. Tel.: +33 174071820.

**Figure S1**


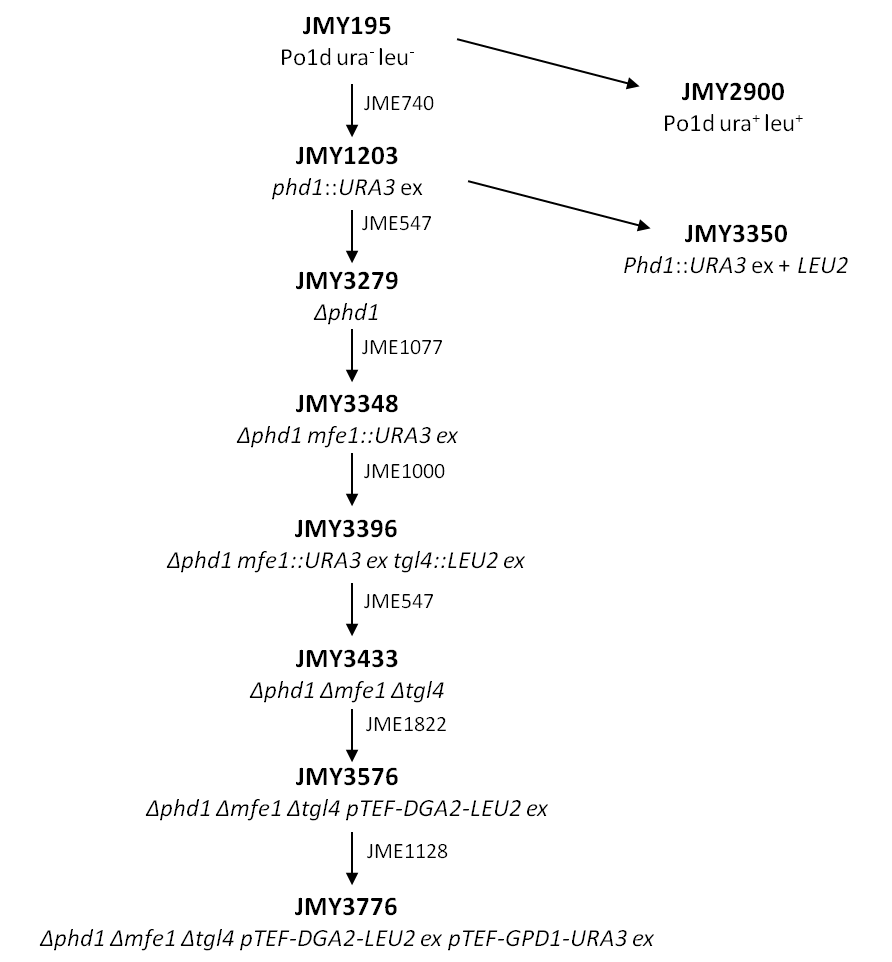


**Figure S1** Schematic representation of strain construction. The auxotrophic strain Po1d (Leu− Ura−) was derived from the French wild-type strain W29. First, *PHD1* was disrupted with the *phd1*::*URA3*ex disruption cassette (JME740) yielding JMY1203 and JMY3350 after *LEU2* complementation. Uracil auxotrophe was restored by marker rescue yielding JMY3279. Second, *MFE1* and TGL4 were disrupted with the *mfe1*::*URA*3ex disruption cassette (JME1077) yielding JMY3348 and the *tgl4*::*LEU2*ex disruption cassette (JME1000) yielding JMY3396. Uracil and leucine auxotrophies were restored by marker rescue yielding JMY3433. Finally, p*TEF*-*DGA2*-*LEU2ex* (JME1822) and p*TEF*-*GPD1*-*URA3ex* (JME1128) overexpression cassettes were introduced yielding JMY3776 (obese ∆*phd1*)*.* For more information about construction, see materials and method section and Table 1.

**Figure S2**

Cell growth in different concentration of propionate


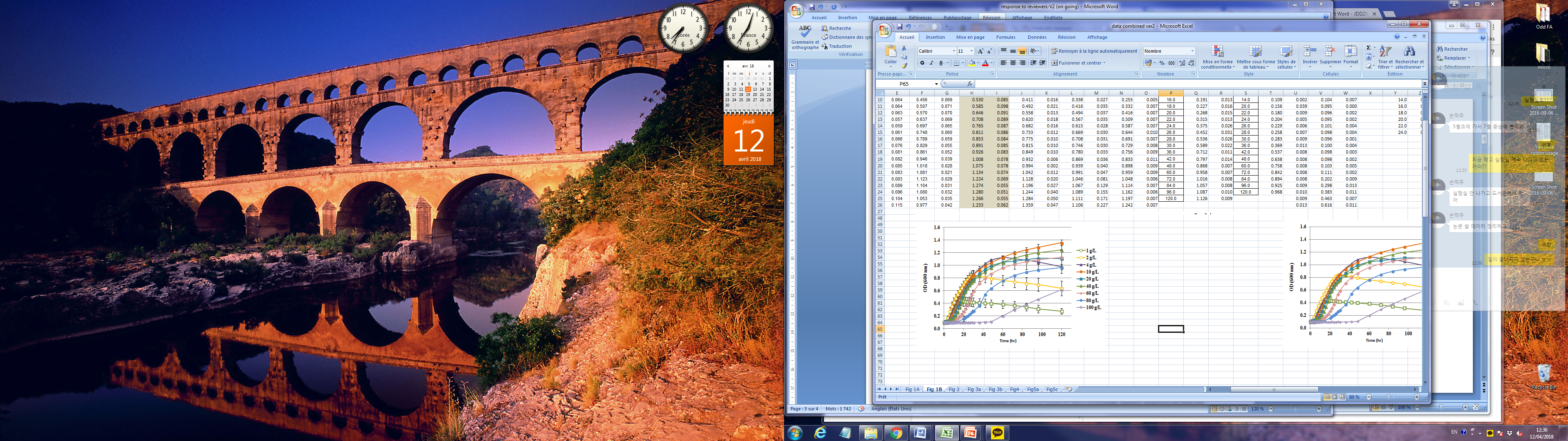


**Figure S3**

GC chromatogram of lipid profiles of JMY2900 (WT) and JMY3776 (obese ∆*phd1*)


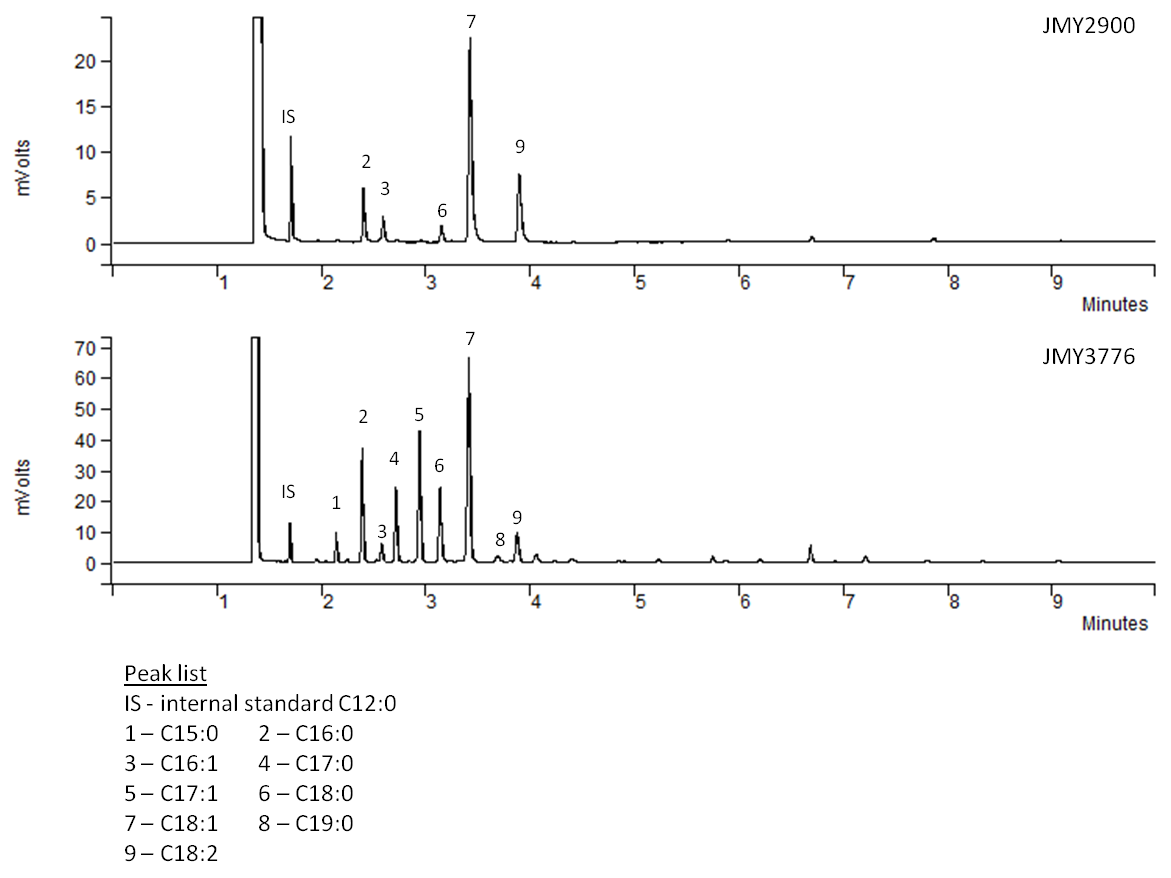


**Table S1**

Growth rate of JMY2900 on glucose and weak acids

|  | Growth rate  (μ_max_, h^-1^) | maximal OD |
| --- | --- | --- |
| Glucose | 0.249 ± 0.076 | 1.447 ± 0.009 |
| Acetate | 0.170 ± 0.024 | 1.020 ± 0.079 |
| L-Lactate | 0.177 ± 0.021 | 0.529 ± 0.028 |
| Propionate | 0.161 ± 0.035 | 1.184 ± 0.066 |

The mean value of three independent experiments is shown and the standard deviation is indicated.

**Table S2**

Growth rate of JMY2900 on propionate depending on concentration

| Propionate  (g/L) | Growth rate  (μ_max_, h^-1^) | maximal OD |
| --- | --- | --- |
| 1 | 0.195 | 0.439 |
| 2 | 0.236 | 0.838 |
| 4 | 0.195 | 1.123 |
| 10 | 0.179 | 1.359 |
| 20 | 0.145 | 1.111 |
| 40 | 0.161 | 1.242 |
| 60 | 0.142 | 1.126 |
| 80 | 0.180 | 0.968 |
| 100 | 0.029 | 0.616 |

**Table S3**

Lipid production of JMY2900 and JMY3350 in YNBD1

| YNBD1 | Biomass (g/L) | Lipid content (%) | | Odd lipids /Total lipids (%) | | Lipids (g/L) | | |
| --- | --- | --- | --- | --- | --- | --- | --- | --- |
|  |  | Total | Odd |  |  | Total | | Odd |
| JMY2900  (WT) | 5.37 ± 0.13 | 7.86 ± 0.13 | 0.14 ± 0.01 | | 1.75 ± 0.02 | | 0.48 ± 0.01 | 0.008 ± 0.001 |
| JMY3350  (WT *Δphd1*) | 5.35 ± 0.15 | 7.43 ± 0.24 | 0.18 ± 0.01 | | 2.36 ± 0.15 | | 0.47 ± 0.01 | 0.011 ± 0.001 |

The mean value of three independent experiments is shown and the standard deviation is indicated.

**Table S4**

Odd chain Fatty acid production in *Y. lipolytica*

| Strain | Substrate | Biomass (g/L) | Lipid content % (w/w) | Odd lipids /Total lipids (%) | Odd lipids (g/L) | Reference |
| --- | --- | --- | --- | --- | --- | --- |
| *wild-type* | Propionate 8 g/L | 3.38 | 25.73 | ND | ND | [1] |
| *wild-type* | Propionate 5 g/L | 2.50 | 27.38 | ND | ND | [2] |
| *wild-type* | Propionate 4 g/L | 3.53 | 8.90 | ~30 | <0.093 | [3] |
| *wild-type* | Glucose 20 g/L  + Propionate 4 g/L | 3.83 | 10.20 | <15 | <0.040 | [3] |
| *wild-type* | Pentadecane 3 g/L  + Rhamnolipids | 2.75 | 17.2 | 44.5 | 0.209 | [4] |
| *wild-type* | Propionate 10 g/L | 2.60 | 7.48 | 34.96 | 0.06 | This study |
| *Δphd1* | Glucose 24 g/L  + Propionate 4 g/L | 4.50 | 8.01 | 46.82 | 0.17 | This study |
| *Obese Δphd1* | Glucose 24 g/L  + Propionate 4 g/L | 5.53 | 24.76 | 41.9 | 0.57 | This study |

ND: not determined

[1] Fontanille P, Kumar V, Christophe G, Nouaille R, Larroche C. Bioconversion of volatile fatty acids into lipids by the oleaginous yeast *Yarrowia lipolytica*. Bioresource Technology. 2012;114:443-9.

[2] Gao R, Li Z, Zhou X, Cheng S, Zheng L. Oleaginous yeast Yarrowia lipolytica culture with synthetic and food waste-derived volatile fatty acids for lipid production. Biotechnology for Biofuels. 2017;10:247.

[3] Kolouchová I, Schreiberová O, Sigler K, Masák J, Řezanka T. Biotransformation of volatile fatty acids by oleaginous and non-oleaginous yeast species. FEMS Yeast Research. 2015;15:fov076.

[4] Matatkova O, Gharwalova L, Zimola M, Rezanka T, Masak J, Kolouchova I. Using Odd-Alkanes as a Carbon Source to Increase the Content of Nutritionally Important Fatty Acids in *Candida krusei*, *Trichosporon cutaneum*, and *Yarrowia lipolytica*. International Journal of Analytical Chemistry. 2017;2017:9.
